# Combined association analysis of interleukin 1-receptor antagonist (*IL-1RN*) variable number of tandem repeat (VNTR) and Haptoglobin 1/2 polymorphisms with type 2 diabetes mellitus risk

**DOI:** 10.1186/s40200-016-0232-z

**Published:** 2016-03-29

**Authors:** Habiba Al-Safar, Wala Kamal, Ahmed Hassoun, Wael Almahmeed, Naushad Rais

**Affiliations:** 1 Khalifa University Center of Biotechnology, Khalifa University of Science, Technology & Research, P.O. Box 127788, Abu Dhabi, United Arab Emirates; 2 Khalifa University Center of Biotechnology, P.O. Box 127788, Abu Dhabi, United Arab Emirates; 3 School of Life Sciences, Manipal University, P.O. Box-345050, Dubai, United Arab Emirates; 4 Dubai Diabetes Centre, Dubai Health Authority, Dubai, United Arab Emirates; 5 Institute of cardiac science, Sheikh Khalifa Medical City, Abu Dhabi, United Arab Emirates

**Keywords:** Genetic polymorphism, Haptoglobin, Interleukin-1 receptor antagonist, Type 2 Diabetes Mellitus

## Abstract

**Background:**

The polymorphism of Interleukin 1 receptor antagonist gene (*IL-1RN*), which encodes a natural antagonist of pro-inflammatory cytokines belonging to IL-1 family and Haptoglobin (*HP*) have been studied in various ethnic groups in association with Type 2 Diabetes Mellitus (T2DM) risk and related complications. However, there was no study available among the Emirati population. Hence, we designed a combined study on *IL-1RN* and *HP* polymorphism to evaluate their association with prevalence of T2DM, related complication and hypertension and also its interaction with obesity status among Emirati population.

**Methods:**

*IL-1RN* and *HP* genotypes were determined in total 487 Emiratis divided in two groups of T2DM case (*n* = 271) and healthy controls (*n* = 215) by polymerase chain reaction (PCR) followed by gel electrophoresis. Gene-gene interaction and polymorphism-obesity interaction were determined by multivariate logistic regression analysis.

**Results:**

We found that the frequencies of IL-1RN*1/*1 and *HP2-2* genotypes were significantly higher in cases than control and were associated with increased T2DM risk with an odds ratio (OR) of 1.60 (95 % CI 1.10–2.36) and 1.63 (95 % CI 1.11–2.64), respectively. The association lack any interaction with obesity status. Associations with occurrences of T2DM related complications and hypertension were not observed.

**Conclusions:**

We report an association of *IL-1RN* and *HP* polymorphism with T2DM risk independent of each other and of obesity status but no association with related complications and hypertension.

**Electronic supplementary material:**

The online version of this article (doi:10.1186/s40200-016-0232-z) contains supplementary material, which is available to authorized users.

## Background

The recent update from 6th edition of the IDF Diabetes Atlas estimated 382 million people to have diabetes worldwide, with dramatic increases seen in countries all over the world including the Middle East North African region (MENA) region in which 34.6 million people, or 9.2 % of the adult population have diabetes. This number is set to almost double to 67.9 million by 2035, which is overwhelmingly due to increased prevalence of Type 2 Diabetes Mellitus (T2DM) in the region (IDF 6th Edition, 2013). More than 50 % diabetic patients had major life threatening complications such as cardiovascular disease (CVD) like coronary heart disease, stroke, retinopathies and nephropathies associated with T2DM [1]. There is increasing evidence that genetic, environment and life style related factors contribute to the T2DM risk. However, genetic susceptibility appears to play a crucial role in the etiology and manifestation of the disease [2–4]. In recent years, numerous studies examining various ethnic groups have demonstrated an association of haptoglobin (*HP)* genotypes, interleukin-1 receptor antagonist protein (IL-1Ra) levels with T2DM [5–7]. Indeed, IL-1Ra had been proposed as one of the potential therapeutic target and diagnostic biomarker of T2DM [8, 9].

HP is a plasma α2-glycoprotein which is synthesized by hepatocytes in response to infection or inflammation and binds free haemoglobin, thus preventing oxidative damage. The *HP* gene is a polymorphic gene giving rise to 3 major genotypes *HP1-1, HP2-1 and HP2-2*. Protein encoded by *HP1-1* genotype is a dimer, however the one encoded by *HP2-1* and *2*–*2* exist as linear and circular polymers, respectively [10]. The frequency of the HP genotypes varies worldwide for example, the frequency of *HP 1–1*, *HP1-2* and *HP2-2* genotype in Western world were 16, 48 and 36 %, respectively [11–13]. *HP* genotypes have been linked to susceptibility to various T2DM related complications especially CVDs [5, 14] because of its involvement in states of oxidation and inflammation.

Interleukin-1 receptor antagonist gene (*IL-1RN*) encodes a naturally occurring competitive inhibitor of IL-1 that binds to the type I receptor and protects human pancreatic cells from IL-1β-induced functional impairment and apoptosis [15, 16]. Recent work has demonstrated that Gastric inhibitory polypeptide (GIP) induced inflammatory and pro-lipolytic response which impairs insulin sensitivity of glucose uptake in human adipocytes is mediated by IL-1α dependent signalling [17]. *IL-1RN* is abundantly expressed in adipose tissue in obese rodents and humans [18]. Serum levels of IL-1Ra are markedly increased in obesity [19, 20] and are considered as predictor of T2DM [20]. The *IL-1RN* gene demonstrates variable number of tandem repeat (VNTR) polymorphism within intron 2 due to an 86 bp sequence repeat. Six distinct alleles have been identified so far which correspond to 1, 2, 3, 4, 5 and 6 repeats of 86 bp sequence [21]. The alleles designated as IL-1RN*1 with 4 repeat and IL-1RN*2 with 2 repeat are most frequently observed in the general populations and the other four alleles are rare. The repeated sequence at polymorphic site may have functional significance as it contains possible binding site for transcription factors [22]. There are not many reports available on the association of this polymorphism with T2DM risk and its interaction with hypertension and obesity status in Arab population. Taken together, we designed the present study on the combined analysis of polymorphisms of *HP* and *IL-1RN* genes in T2DM and related complications and its interaction with hypertension and obesity status in Emirati population, which largely remained unexplored with regards to genetic association studies on T2DM.

## Methods

### Subjects and sample collection

This study was conducted on four hundred and eighty seven (*n* = 486) unrelated Emiratis who were identified during their routine visit to a diabetes clinic in Dubai and Abu Dhabi, United Arab Emirates and agreed to take part in this study. The case group of 271 T2DM had mean age of 58 ± 12 years and consisted of 60 % females. The control group consisted of 215 healthy individuals with mean age 45 ± 16 years and 68.37 % female. All participants gave their informed consent in writing according to Helsinki’s declaration (revision, 2008). There were several inclusion and exclusion criteria used in this study; Inclusion criteria: UAE National, diagnosed with T2DM and healthy individuals, able to give consent and above 18 years old. Exclusion Criteria: Non-UAE national, pregnant female, not be able to consent and less than 18 years old. The Ethics committees of the Dubai Health Authority and Sheikh Khalifa Medical City (SKMC) research ethics committee in the United Arab Emirates approved the study.

After taking an informed consent, each participant was asked to deliver 1 ml of saliva into an Oragene kit OGR-500 (DNA Genotek, Ottwa, Canada) collection tube. The saliva samples were preserved for DNA extraction and genotyping later. The genomic DNA was extracted from saliva using prepITTM•L2P (DNA Genotek, Ottwa, Canada) DNA extraction kit in accordance with the manufacturer’s instructions.

In addition, a clinical assessments and validated lifestyle questionnaire were completed at the clinic to study any correlation between lifestyle variables and genetic variation. An individual was classified as T2DM if the subject was (1) diagnosed with T2DM by a qualified physician, (2) on a prescribed drug treatment regimen for T2DM and (3) returned biochemical test results of a fasting plasma glucose level of at least 126 mg/dl based on the criteria outlined by the World Health Organization (WHO) consultation group report [23]. The individuals with BMI more than 30 were considered obese and those with a BMI less than 30 were grouped into the non-obese population. According to JNC 8 classification all the individuals with blood pressure more than 140/90 mmHg were considered hypertensive [24].

### PCR based genotyping for *HP 1/2* polymorphism

The *HP* genotypes were determined by polymerase chain reaction (PCR) followed by agarose gel electrophoresis. PCR primers, reaction and cycling conditions were performed as described previously by Koch et al. [25]. Briefly, 20 μL reaction mixture contained 1X PCR buffer (as suggested by supplier), 2 U of *Taq* polymerase (Qiagen, Hilden, Germany), 25–100 ng of DNA, 200 μM each dNTPs and all four primers at final concentrations of 0.8 μM each. After initial denaturation at 95 °C for 2 min, the two-step thermocycling procedure consisted of denaturation at 95 °C for 1 min, annealing and extension at 69 °C for 2 min, repeated for 35 cycles, and followed by a final extension at 72 °C for 7 min. All PCR reactions took place in the Thermo cyclers GeneAmp PCR systems 9700 (Applied Biosystems, Foster City, CA). For genotype determination the PCR products were separated on 1 % agarose gels and visualized by ethidium bromide staining.

### *IL-1RN* gene intron 2 variable number tandem repeat polymorphism genotyping

The *IL-1RN* gene intron 2 VNTR polymorphism was analyzed as previously described [26] under the following PCR conditions: denaturation at 94 °C for 5 min, followed by 35 cycles at 94 °C for 30 s, 55 °C for 30 s, 72 °C for 45 s, and a final extension step at 72 °C for 7 min in Veriti 96 well Thermo Cycler (Applied Biosystems, Foster City, CA). The reaction was performed in 25 μL reaction mixture containing 1X PCR buffer, 1.25 U of *Taq* polymerase (Qiagen, Hilden, Germany), 25–100 ng of DNA, 200 μM each dNTP and primers at final concentrations of 1 μM each.

### Statistical methods

Statistical analyses were performed using STATA version 13 (STATA Corp., TX, USA). All continuous variables were expressed as the mean ± SD or as percentages for categorical variables. The genotype frequencies were tested for the Hardy-Weinberg equilibrium using a χ2 test. Analysis of statistical power was done using the online Genetic Power Calculator. The Fisher exact test was used to compare the continuous variables between T2DM and healthy groups with respect to genotype distributions and allele frequencies. The Student’s t test was used to compare the continuous variables between the T2DM and healthy groups. Fisher exact confidence intervals were drawn for relative risk estimates and calculated with a 95 % confidence interval. The following gene transmission models were considered for haptogloin: (1) recessive effect (TT Vs CC *+* CT), (2) a dominant effect (*CC* Vs CT *+* TT) and (3) additive effect of T allele. The association was evaluated by chi square test followed by logistic regression. A *p* value of < 0.05 was considered for statistical significance. The interaction of *HP* genotypes (*1*–*1*, *1*–*2*, *2*–*2*), *IL-1RN* genotypes (*1/*1 and *2/*2) with each other and with obesity and hypertension in relation to T2DM risk was also evaluated with logistic regression using the two by four tables.

## Results

The demographic characteristics, clinical and biochemical parameters of T2DM patients and healthy control patients are described in Table 1. The mean age and male/female ratios significantly differed in the affected individuals and healthy controls. The cases were significantly older than the controls (58 ± 12 Vs 45 ± 16, *p* < 0.001), their mean BMI, lipid profile and blood pressure levels were also significantly different (Table 1). No significant differences were observed in these parameters between different *IL-1RN* genotypes, however HB1Ac level were higher in *HP1-2* + *2-2* genotypes than the *HP1-1* homozygotes (Table 2). The genotype and allele frequency distribution for various alleles has been provided as Additional file 1.

**Table 1:** Demographic, clinical and biochemical characteristics of T2DM patients and healthy subjects in the studied population

	T2DM patients *n* = 271	Healthy subjects *n* = 215	*p*
Age (years)	58 ± 12	45 ± 16	<0.0001
Male: Female Ratio	110:161	69:146	0.05
BMI	31.99 ± 6.3	29.97 ± 6.14	0.0009
Fasting Glucose (mmol/L)	8.99 ± 7.23	5.63 ± 7.23	<0.0001
Systolic Blood Pressure (mmHg)	129.97 ± 17.24	122.50 ± 15.65	<0.0001
Diastolic Blood Pressure (mmHg)	70.0 ± 11.0	71.35 ± 11.45	0.6197
HBA1c (mmol/L)	7.6 ± 1.6	5.59 ± 0.56	<0.0001
Cholesterol (mmol/L)	4.14 ± 1.05	4.49 ± 0.96	0.0001
Triglycerides (mmol/L)	1.46 ± 0.83	1.18 ± 0.76	0.0041
HDL (mmol/L)	1.22 ± 0.49	1.31 ± 0.51	0.1316
LDL (mmol/L)	2.30 ± 0.91	2.66 ± 0.93	0.0008

*BMI* body mass index, *HDL* high-density lipoprotein, *LDL* Low-density lipoprotein

**Table 2:** Clinical and biochemical characteristics in different *HP* and *IL-1RN* genotypes

	HB1Ac (mmol/L)	Cholesterol (mmol/L)	Triglycerides (mmol/L)	HDL (mmol/L)	LDL (mmol/L)	BMI Kg/m^2^	SBP mmHg	DBP mmHg
*HP*								
1-1	6.54 ± 1.06	4.30 ± 1.09	1.27 ± 0.893	1.34 ± 0.84	2.54 ± 1.02	30.1 ± 6.62	123.6 ± 17.6	70.61 ± 11.16
1-2 + 2-2	7.15 ± 1.74	4.25 ± 1.03	1.39 ± 0.801	1.24 ± 0.17	2.41 ± 0.92	31.2 ± 6.40	127 ± 16.7	70.92 ± 11.63
* p* ^a^	0.025	0.749	0.3417	0.067	0.368	0.173	0.12	0.836
*IL-1RN*								
*1/*1	7.36 ± 4.86	4.31 ± 1.06	1.36 ± 0.82	1.26 ± 0.49	2.48 ± 0.97	30.8 ± 6.27	126.5 ± 16.9	70.0 ± 11.8
*1/*2	7.11 ± 1.91	4.15 ± 0.99	1.39 ± 0.83	1.24 ± 0.56	2.29 ± 0.87	31.6 ± 6.54	127.6 ± 16.8	72.5 ± 10.6
* p* ^a^	0.663	0.216	0.773	0.751	0.106	0.232	0.548	0.045

Data show mean ± SD; ^a^student’s t-test, SBP: Systolic Blood Pressure, DBP: Diastolic Blood Pressure

### Association of *IL-1RN* VNTR and *HP* 1/2 polymorphism with T2DM risk regardless of obesity status

The results of logistic regression analysis for three gene transmission models for *HP 2* allele showed that odd ratios were significant only in dominant model (*HP2-2* + *1-2 Vs 1–1*, [OR = 1.63 (95%CI 1.11–2.64, *p* = 0.0442)] (Table 3) demonstrating *HP 1–2* or *2*–*2* genotype as a risk factor for T2DM occurrence. Because of the absence of homozygote for other alleles, in case of *IL-1RN* VNTR polymorphism the association with T2DM risk was analyzed only IL-1RN*1/*1 Vs all other observed genotypes in which IL-1RN*1/*1 genotypes was found significantly associated with T2DM risk [OR 1.60 (95 % CI 1.10–2.36), *p* = 0.0192] as suggested by logistic regression analysis.

**Table 3:** Analysis of association of T2DM with *HP* and *IL-1RN* genotypes in Emirati population

*HP* Gene transmission	T2DM		*p* ^a^	Logistic regression		
Recessive model	Yes (1)	No (0)		OR	95 % CI	*p*
2-2	132	92	0.2067	1.2606	0.8795–1.8066	0.2073
1-1 + 1-2	140	123				
Dominant model						
2-2 + 1-2	235	171	0.0442	1.63	1.11–2.6399	0.0447
1-1	37	44				
Additive						
1-1 (0)	37	44	0.11648			
1-2 (1)	103	79				
2-2 (2)	132	92				
*IL-1RN* Genotypes						
*1/*1	200	138	0.019	1.60	1.10–2.36	0.0192
*1/*2 + others	69	76				

^a^Overall model

### Evaluation of interaction between two genetic polymorphisms and with obesity and hypertension in relation to T2DM risk

Upon evaluation of interaction between *IL-1RN* and HP polymorphisms in T2DM, it was found that there was no significant interaction between *HP 1–2* or *2–2* genotypes with IL-1RN *1/*1 genotype as analyzed by multivariate logistic regression analysis (Odd ratio of interaction variable 0.8064 [95 % CI 0.2569—2.5320, *p* = 0.7125]) (Table 4) suggesting the association of these two polymorphisms with T2DM risk was independent of each other. These associations were also found independent of obesity status (Table 5). The allele and genotype frequency distribution of *IL-1RN* and *HP* polymorphism in T2DM patients with or without hypertension and obesity also did not show any significant differences in genotype and allele frequencies between the subgroups (Table 6).

**Table 4:** Evaluation of gene-gene Interaction between *HP* and *IL-1RN* polymorphisms in T2DM

*HP* genotypes	*IL-1RN* genotypes	T2DM		*p* ^a^	Logistic regression		
		Yes (1)	NO (0)		OR	95 % CI	*p*
1-2 + 2-2 (1)	*1/*1	170	111	0.0223	2.0800	0.7167–6.0364	0.1779
	Others	63	69		1.6957	0.6362–4.5193	0.2910
1-1 (0)	*1/*1	28	25		0.8064	0.2569–2.5320	0.7125
	Others	7	13				

^a^Overall model

**Table 5:** Evaluation of interaction of obesity status with *HP* and *IL-1RN* polymorphisms and T2DM association in Emiratis

Obesity status	Genotypes	T2DM		*p* ^a^	Logistic regression		
		Yes	No		OR	95 % CI	*p*
BMI > 30	*HP*1-2 + 2-2	134	76	0.0675	1.4399	0.6986–2.96767	0.3232
	*HP*1-1	19	12		1.9792	0.7450–5.2575	0.1708
BMI < 30	*HP*1-2 + 2-2	92	79		0.7734	0.2678–2.233	0.6348
	*HP*1-1	16	20				
BMI > 30	*Il-1RN* *1/*1	110	56	0.0989	0.7705	0.4435–1.3385	0.3548
	*IL-1RN* *1/*2 + others	40	25		1.1022	0.5598–2.1702	0.7783
BMI < 30	*IL-1RN* *1/*1	85	76		1.5934	0.7079–3.5866	0.2604
	*IL-1RN* *1/*2 + others	45	31				

^a^Overall model

**Table 6:** Allele and Genotype frequencies of *IL-1RN* VNTR and *HP* 1/2 polymorphisms in T2DM patients with or without complications, hypertension and obesity

	Complications		*p* ^a^	Hypertension		*P* ^a^	Obesity		*p* ^a^
	*N* (%)			*N* (%)			*N* (%)		
	Yes	No		Yes	No		Yes	No	
*IL-1RN*									
*1/*1	77 (74.0 %)	79 (68.7 %)	0.455	130 (73.9 %)	65 (71.4 %)	0.666	110 (73.3 %)	85 (65.4 %)	0.163
*1/*2 + others	27 (26.0 %)	36 (31.3 %)		46 (26.1 %)	26 (28.6 %)		40 (26.7 %)	45 (34.6 %)	
Allele Frequencies									
*1	0.87	0.83	0.1573	0.87	0.86	0.686	0.87	0.83	0.1963
*2	0.10	0.14		0.10	0.11		0.10	0.12	
*HP*									
1-1	15 (14.3 %)	13 (11.6 %)	0.2885	28 (15.9 %)	9 (9.9 %)	0.196	19 (12.4 %)	16 (14.8 %)	0.5851
1-2 + 2-2	90 (85.7 %)	99 (88.4 %)		148 (84.1 %)	82 (90.1 %)		134 (87.6 %)	92 (85.2 %)	
Allele frequencies									
1	0.33	0.30	0.5353	0.34	0.30	0.382	0.30	0.37	0.1562
2	0.67	0.70		0.66	0.70		0.70	0.63	

^a^Fisher exact test

### *HP* and *IL-1RN* polymorphism and complications in patients with T2DM

The allele and genotype frequencies in T2DM patients with complications and without complications were not significantly different (Table 6) thus suggesting no association of these polymorphisms with the occurrence of T2DM related complications.

## Discussion

T2DM is characterized by progressive β-cell failure and insulin resistance in which the inflammation and resulting immune response are thought to play a pathogenic role for disease development [27]. Pro-inflammatory cytokines like IL-1β induces apoptosis in insulin-producing β-cells [28] while CCL2 and TNFα are known to impair insulin signalling [29, 30], and therefore causing insulin resistance [31]. The present study focuses on two important genes *HP* and *IL-1RN*, the product of which are associated with inflammation and immune response, respectively. The active HP protein encoded by *HP1-1* genotype induces the production of several cytokines and anti-inflammatory mediators when complexed with haemoglobin thus preventing oxidative damage through mechanisms including stabilization of the heme iron within haemoglobin [32, 33]. IL-Ra protein acts as an anti-inflammatory cytokine which is the natural inhibitor of pro-inflammatory cytokines such as IL-1β. Larsen et al. [34] demonstrated that administration of exogenous IL-1Ra in T2DM patients could preserve endogenous insulin production and attenuate inflammation. Hence, the genetic polymorphism in these genes may be expected to be associated with T2DM in Emirati population. We designed the study to analyze (i) the association of *IL-1RN* VNTR and *HP* 1/2 polymorphism with T2DM, related complication and hypertension individually; (ii) the association’s potential interaction with each other and (iii) with obesity as it is one of the life style related factor associated with T2DM independently.

We observed a high frequency of Il-1RN*1/*1 in our population which was only marginally associated with T2DM (*p* = 0.036) (See Additional file 1) and there was no association with either of the allele. Because of the absence of *2/*2 or other mutant homozygote the comparison was made only IL-1RN*1/*1 Vs *1/*2 plus all other genotypes. Previously, Bid et al. [7] also demonstrated a high frequency of IL-1RN*1/*1 genotypes in Indian T2DM patients. Moreover, allele IL-IRN *2 has previously been demonstrated to be associated with enhanced in vitro production of IL-IRa by granulocytemacrophage colony-stimulating factor (GM-CSF)-stimulated monocytes [35]. In an in-vivo study [36], patients with type 1 diabetes carrying IL-1RN*1/*1 genotype had 30 % lower levels of plasma IL-1Ra compared with levels in patients carrying the IL1RN*1/*2 genotype further suggesting an enhancing effect of IL1RN*2 allele on IL-1Ra circulating levels in healthy carriers. We also observed a significantly higher risk of T2DM in individuals carrying *IL-IRN* *1/*1 genotype (OR 1.5963 (95 % CI 1.0792–2.3612, *p* = 0.0192) (Table 3). We identified marginally significant differences in the *HP* genotype and allele frequencies between the T2DM patients and healthy subjects. The frequencies of *HP2-2* genotype and *HP 2* allele were higher in T2DM group as compared to control (48.5 % Vs 33.8 %) (see Additional file 1) and it carries significantly higher risk of T2DM but only in dominant model (Table 3). The *HP* genotype frequencies in this study were similar to those reported previously [11–13] in Caucasians. Several previous studies have investigated the effect of the *HP* polymorphism on T2DM risk. A recent study by Adams et al. [5] reported that *HP2-2* genotype was associated with T2DM risk in European American. A second report by Quaye et al. [37] found that the *HP 2–2* phenotype was a risk factor for T2DM in a Ghana population. Upon literature exploration about the possible cause of this association we found that different *HP* allele may lead to different circulating levels of HP protein [38, 39]. High circulating HP protein as result of gene duplication (*HP2*) has been associated with metabolic syndrome, high blood pressure, and elevated glucose [40] and this could be possible explanation of the association of the *HP* polymorphism with T2DM risk. However, we did not find any correlation of *HP* genotypes with diabetes dependent hypertension (Table 6).

In order to investigate gene-gene interaction, we analyzed the combined effect of *HP* and *IL-1RN* polymorphism on, and their possible association with, T2DM. Multivariate logistic regression analysis results were obtained to evaluate the interaction which failed to show any interaction between two polymorphism (Table 4), suggesting these polymorphisms are associated with T2DM independent of each other. In addition, we also evaluated the effect of obesity status and possible interaction with these two gene polymorphism as obesity is one of the risk associated with T2DM. Moreover, in some studies the associations of SNPs in some of the T2DM candidate genes have previously been shown to be modulated by obesity status [41]. However, we did not observe any interaction of obesity status with *HP* and *IL-1RN* polymorphism with regard to association with T2DM risk hence, it is unlikely to have cofounded the *HP* and *IL-1RN* associations describe here.

We also evaluated the effect of these polymorphisms on the incidences of T2DM related complications and hypertension which may be expected as variable circulating levels of HP and IL-1Ra in different genotypes as described elsewhere may affect the oxidative and inflammatory status in the body. To our surprise, we did not observe any association of *HP* and *IL-1RN* genotypes and allele frequencies with T2DM related complications and hypertension (Table 6). One of the possible causes of no association may be due to small sample size in the subgroups created based on the absence and presence of any of the complications, resulting in low statistical power for analysis. Moreover, we took the different complications like CVD, stroke, nephropathies and retinopathies all together in the single group for the reason of not having enough samples in individual subgroups. Therefore, this study requires further confirmation with large sample size and all the complications needed to be analyzed individually. We also analyzed the association of different biochemical and clinical parameters of T2DM patients with *HP* and *IL-1RN* genotypes; there was lack of association with any of these parameters except that the HBA1c levels were significantly higher in *HP 1–2* and *HP2-2* genotypes (*p* = 0.025) (Table 2).

## Conclusion

This is the first report on the association of *IL-1RN* and *HP* with T2DM risk in Emirati population. This association is independent of obesity status. We did not find any association of these polymorphisms with T2DM related complications however; the levels of HBA1c were significantly higher in *HP 1–2* and *HP2-2* genotypes. There was no association of these genotypes with hypertension probably because of small sample size, hence require further confirmation.

### Ethics approval and consent to participate

The Ethics committees of the Dubai Health Authority and Sheikh Khalifa Medical City (SKMC) research ethics committee in the United Arab Emirates approved the study. Written informed consent was obtained from all cases and controls.

### Availability of data and materials

Summary data are available from the corresponding author as appropriate for meta-analyses.

## Additional file

Additional file 1: Table S1. Allele and Genotype frequencies of *IL-1RN* VNTR and *HP*1/2 polymorphisms in T2DM patients and healthy subjects regardless of obesity status. (DOCX 12 kb)

## Abbreviations

BMI: body mass index
DNA: deoxyribonucleic acid
GWAS: genome-wide association study
HLD: high density lipoprotein
IDF: International Diabetes Foundation
LDL: low density lipoprotein
SNP: single nucleotide polymorphisms
T2DM: Type 2 Diabetes Mellitus
TG: Triglyceride
