# Supplementary material for: Combined association analysis of interleukin 1-receptor antagonist (IL-1RN) variable number of tandem repeat (VNTR) and Haptoglobin 1/2 polymorphisms with type 2 diabetes mellitus risk
Source: J Diabetes Metab Disord. 2016 Mar 29;15:10. doi: 10.1186/s40200-016-0232-z (PMC4812649; doi:10.1186/s40200-016-0232-z)
Supplement: Additional file 1: Table S1. — Allele and Genotype frequencies of IL-1RN VNTR and HP1/2 polymorphisms in T2DM patients and healthy subjects regardless of obesity status. (DOCX 12 kb) [file 40200_2016_232_MOESM1_ESM.docx]

Additional file 1: Table S1: Allele and Genotype frequencies of *IL-1RN* VNTR and *HP*1/2 polymorphisms in T2DM patients and healthy subjects regardless of obesitystatus

|  | **T2DM patients**  **N (%)** | **Healthy subjects**  **N (%)** | ***p***^†^ |
| --- | --- | --- | --- |
| ***IL-1RN* Genotypes** | | | |
| *1/*1 | 200 (72.4%) | 139(64.7%) | **0.036** ^††^ |
| *1/*2 | 61 (22.5%) | 62(28.8%) |  |
| *1/*3 | 5 (1.8%) | 14(6.5%) |  |
| *1/*4 | 3 (1.1%) | 0 |  |
| *3/*3 | 2 (0.7%) | 0 |  |
| Allele Frequencies |  |  |  |
| *1 | 0.865 | 0.823 | 0.0736 ^†††^ |
| *2 | 0.112 | 0.144 |  |
| *3 | 0.017 | 0.033 |  |
| *4 | 0.006 | 0 |  |
| ***HP* Genotypes** | | | |
| 1-1 | 37(13.6%) | 44(20.5%) | **0.049**^††††^ |
| 1-2 | 103(37.9%) | 79(36.7%) |  |
| 2-2 | 132(48.5%) | 92(33.8%) |  |
| Allele frequencies |  |  |  |
| 1 | 0.33 | 0.39 | **0.043** |
| 2 | 0.67 | 0.61 |  |

^†^Fisher exact test; ^††^*1/*1 Vs all other genotypes; ^†††^*1 vs others; ^††††^1-1 Vs 1-2+2-2
